# Development of Piezoresistive Micropressure Sensor Based on Grooved Diaphragm with Back Peninsulas and Trenches

**DOI:** 10.3390/mi17080968

**Published:** 2026-08-16

**Authors:** Peicang Chen, Lei Guo, Jiahao Feng, Chenxi Li, Yan Liu, Weidong Wang

**Affiliations:** 1 School of Mechano-Electronic Engineering, Xidian University, Xi’an 710071, China; 22043110443@stu.xidian.edu.cn (P.C.); 23041212602@stu.xidian.edu.cn (L.G.); 25041212758@stu.xidian.edu.cn (J.F.); 25041212558@stu.xidian.edu.cn (C.L.); 2 China Electronics Technology Group Corporation No. 58 Research Institute, Wuxi 214035, China

**Keywords:** micropressure sensor, sensitivity–nonlinearity interaction, diaphragm design, RBFNN-based generator

## Abstract

To validate the effectiveness of the grooved diaphragm with back peninsulas and trenches (GDPT) and the radial basis function neural network (RBFNN)-based dimension generator in the development of a 1 kPa piezoresistive micropressure sensor, this paper presents a comprehensive investigation into the design, fabrication, and characterization of the anticipative sensor prototype. The GDPT achieves favorable sensing stress and reduced deflection, enabling a favorable trade-off between sensitivity and nonlinearity; the RBFNN-based generator efficiently determines practicable dimensions for the complex structure, offering a significant improvement over the conventional trial-and-error process. Characterization results demonstrate that the fabricated sensor achieves sensitivity of 13.01 mV/(V·kPa) and nonlinearity of 0.26% FS within the pressure range of 0–1 kPa, in good agreement with the preset design target. This work verifies the validity of the proposed approach and offers a holistic methodology for developing high-performance piezoresistive sensors.

## 1. Introduction

The growing demand for micropressure measurement in advanced manufacturing, precision medicine, and aerospace has positioned the relevant detection system as a critical enabler in biological monitoring, ventilator control, sealing inspection, and other applications [1,2]. Piezoresistive micropressure sensors based on MEMS technology, characterized by their simple structure, convenient signal conditioning, mature fabrication, and good batch consistency, have become some of the most popular devices in this field [3]. However, conventional sensing diaphragm architectures often face a significant challenge under micropressure conditions: reducing the diaphragm thickness can improve the sensitivity but also leads to drastically increased deflection or aggravated nonlinearity, whereas increasing the thickness can suppress nonlinearity but degrades sensitivity [4]. This inherent interaction necessitates ongoing efforts devoted to structural design and the accompanying dimensional determination.

Generally, the sensing stress in the piezoresistor region for sensitivity and the maximum diaphragm deflection for nonlinearity are set as the key targets in diaphragm design [5,6]. Various engineered components have been introduced into the top and bottom layers of the conventional flat diaphragm (FD) to achieve both stress concentration and partial stiffening [7,8]. The grooves on the top layer, forming a grooved diaphragm (GD), can thin the diaphragm edge to create stress concentration regions for improved sensitivity, alongside an undesired increase in deflection causing pronounced nonlinearity [9]. Additional peninsulas or beams on both the top and bottom layers can stiffen the structure to limit the deflection to an acceptable level, but the resulting promotion of stress energy is unsatisfactory for highly sensitive sensors. Therefore, extensive research continues to focus on improving the diaphragm configuration. Hao et al. introduced a widened root to the cross-beam design to improve the stress uniformity for the piezoresistor region without greatly affecting the structural deflection and dynamic characteristics [10]. The peninsula can be shaped into an inverted trapezoid and slotted to effectively direct the stress energy into narrow regions [11,12]. When bottom elements are introduced, the structural configurations become more diverse. Back bosses, ribs, and peninsulas can all contribute to improving the sensing behavior of the diaphragm, and the collaboration between these dual-layer elements will provide further promotion [13,14]. For example, the combination of front ridges and back peninsulas, as proposed by Xu et al., realizes a distinct high-stress region for the piezoresistor while limiting the structural deflection to address the nonlinearity issue [15]. Introducing engineered elements into the diaphragm has become a powerful approach to designing high-performance piezoresistive micropressure sensors.

Following the diaphragm’s structural design, the determination of dimensional parameters presents a challenge due to the structural complexity induced by additional engineered elements. This becomes an extremely difficult task when using conventional theoretical formulas to describe the structural response behavior. Consequently, early empirical studies pursued an acceptable set of dimensions based on trial-and-error iterations. Some efforts have also attempted to fit the FEA results to a polynomial expression, providing a possible basis for mathematical optimization methods [16]. More effective solutions are achieved with the involvement of neural network techniques in sensor structural design [17]. Many different black-box models have been established to predict the response behavior of sensing structures. These predictive models can serve as a reliable foundation for optimizing the dimensional parameters with a genetic algorithm (GA) or the particle swarm optimization (PSO) algorithm [9]. Favorable design outcomes have been demonstrated in many MEMS devices, such as resonators, accelerometers, ultrasonic transducers, and pressure sensors [18,19,20]. Additionally, some further work involves employing neural network models to directly output the structural dimensions according to predefined sensing responses. Several simple sensing structures, such as the cross-beam-mass configuration for accelerometers and the FD for pressure sensors, have been employed as validation subjects to demonstrate the feasibility of this generation approach [21,22].

Motivated by the design strategy of sensing diaphragms and the use of neural network models for dimensional determination, we have developed a grooved diaphragm with back peninsulas and trenches (GDPT) to achieve concentrated sensing stress and limited structural deflection, and we have implemented a radial basis function neural network (RBFNN)-based dimension generator to produce practicable dimensional parameters according to the predefined response behaviors [23]. In this work, experimental application and validation using a 1 kPa micropressure sensor prototype are conducted to demonstrate the effectiveness of the previously proposed scheme. Considering the expected sensitivity and nonlinearity, the corresponding stress in the piezoresistor region and the required deflection are set and input into the dimension generator to obtain critical structural dimensional parameters. After the necessary fine-tuning, a sensor chip is fabricated and packaged. The obtained prototype is leveraged to verify the feasibility of the proposed strategies. The rest of this paper is organized as follows. Section 2 elaborates on the design of the 1 kPa pressure sensor, and the fabrication, packaging, and characterization of the sensor prototype are presented in Section 3. Finally, the paper is summarized in Section 4.

## 2. Pressure Sensor Design

### 2.1. Principle and Rules

**Figure 1:**
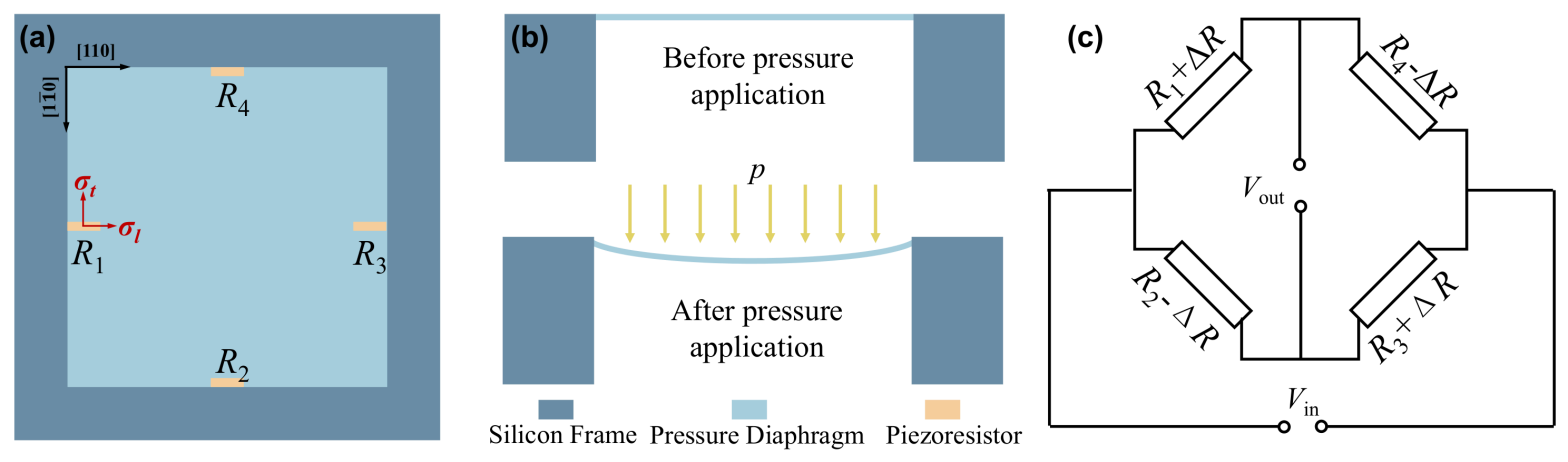
(**a**) Piezoresistive pressure sensor. (**b**) Structural deflection. (**c**) Wheatstone bridge.

Key metrics for evaluating the performance of micropressure sensors include sensitivity and nonlinearity. As shown in Figure 1, a pressure sensor usually employs a circular-, square-, or rectangular-shaped diaphragm to transform the input pressure into a stress field. Four piezoresistors are set on the diaphragm and form a Wheatstone bridge. In the absence of applied pressure, the resistances of all four resistors are ideally equal and the bridge outputs no voltage. When the diaphragm experiences a certain pressure, the accompanying stress causes lattice distortion in the silicon crystal and changes the resistance of the four piezoresistors.

The resistances of two piezoresistors increase and those of the other two decrease, which renders the bridge unbalanced, yielding an output voltage *Vo*. According to the piezoresistive effect of silicon piezoresistors, this voltage is primarily determined by the difference between the longitudinal stress (*σ*_*l*_, along the length direction of the piezoresistor) and the transverse stress (*σ*_*t*_, along the width direction of the piezoresistor) within the piezoresistors, which is presented as [3]

(1) $$V_{o}=\frac{V_{i}}{2}\pi_{44}\left(\sigma_{l}-\sigma_{t}\right)$$

Here, *V_i_* denotes the stimulating voltage of the bridge, and *π*_44_ is the shear piezoresistive coefficient. Generally, the piezoresistors on a diaphragm are placed at high-stress regions in order to maximize the resistance variation and thereby the sensor sensitivity. However, the stress difference within the piezoresistive region is not uniformly distributed, and the value at a single point alone is not sufficient to evaluate the sensitivity. Therefore, the output voltage is often calculated by integrating the stress difference over the piezoresistive region, and Equation (1) can be rewritten as [24]

(2) $$V_{o}=\frac{V_{i}}{2}\pi_{44}\int_{l0}^{l1}\left(\sigma_{l}-\sigma_{t}\right)dx$$

where *l*_0_ and *l*_1_ denote the starting and ending points of the piezoresistive region. In order to enhance the output voltage, it is necessary to increase this stress difference through the optimized design of the sensing diaphragm.

As for the sensor nonlinearity, there are multiple possible contributing factors: geometric nonlinearity, physical nonlinearity, circuit nonlinearity, and process nonlinearity. At the structural design stage, geometric nonlinearity is a significant factor correlated with the diaphragm deflection, and the remaining ones are all inherent deviations that are weakly correlated with the structural design. To achieve low nonlinearity, the diaphragm must conform to small deflection theory, which requires that the maximum deflection does not exceed one fifth of the diaphragm’s thickness [25].

Thus, the design target for a piezoresistive micropressure sensor has been established. It is necessary to strategically introduce engineered components to achieve stress concentration in order to promote the sensor’s sensitivity and limit the deflection to less than one fifth of the diaphragm’s thickness under full-scale loading to ensure linearity.

### 2.2. Design and Analysis of New Diaphragm

**Figure 2:**
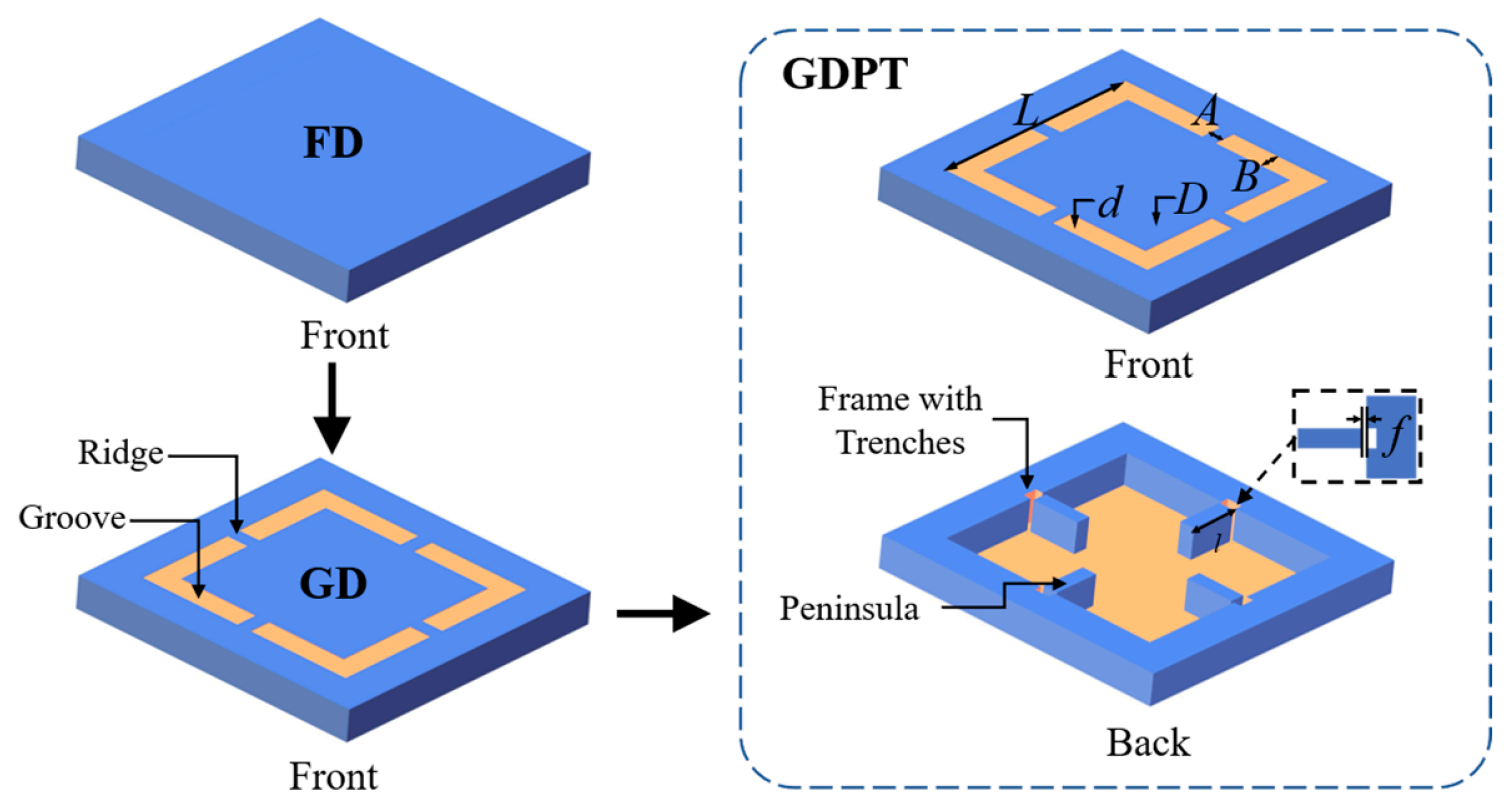
The design of the GDPT for a micropressure sensor.

**Figure 3:**
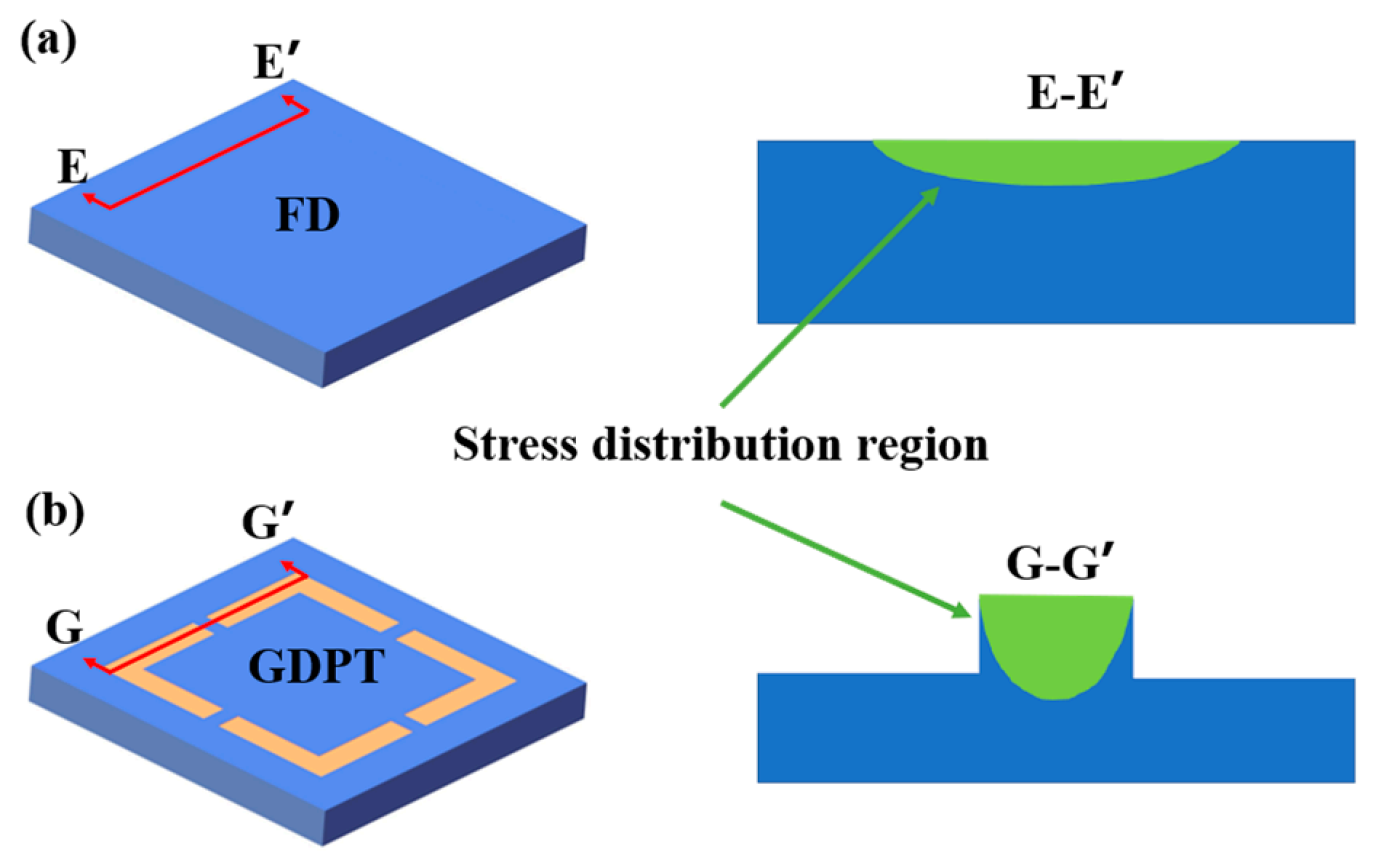
Stress concentration in (**a**) the FD and (**b**) the GDPT.

To address the inherent constraint between sensitivity and linearity in piezoresistive micropressure sensors, we start from the conventional square FD structure and progressively introduce engineered components for stress concentration and local stiffness modulation. As shown in Figure 2, the FD features no additional structures, serving as the benchmark reference in the design; the GD adds four L-shaped grooves at the diaphragm edges to create local thinning and stress concentration for sensitivity improvement; the proposed GDPT further improves the GD by introducing back peninsulas and trenches. The four back peninsulas are positioned beneath the front ridges, with a maintained gap from the frame. This arrangement enhances the existing stress concentration of the GD, and the trenches in the frame can further elevate the sensing stress. As indicated in Figure 3, the width of the stress distribution region in the GDPT is much smaller than that in the FD, and the maximum stress in the new diaphragm exceeds that in the FD under the same pressure load [18,26]. Moreover, the introduced back peninsulas can locally enhance the diaphragm stiffness, thereby mitigating the ballooning effect in diaphragm deformation, which helps to reduce the structural deflection and limit the geometric nonlinearity.

**Figure 4:**
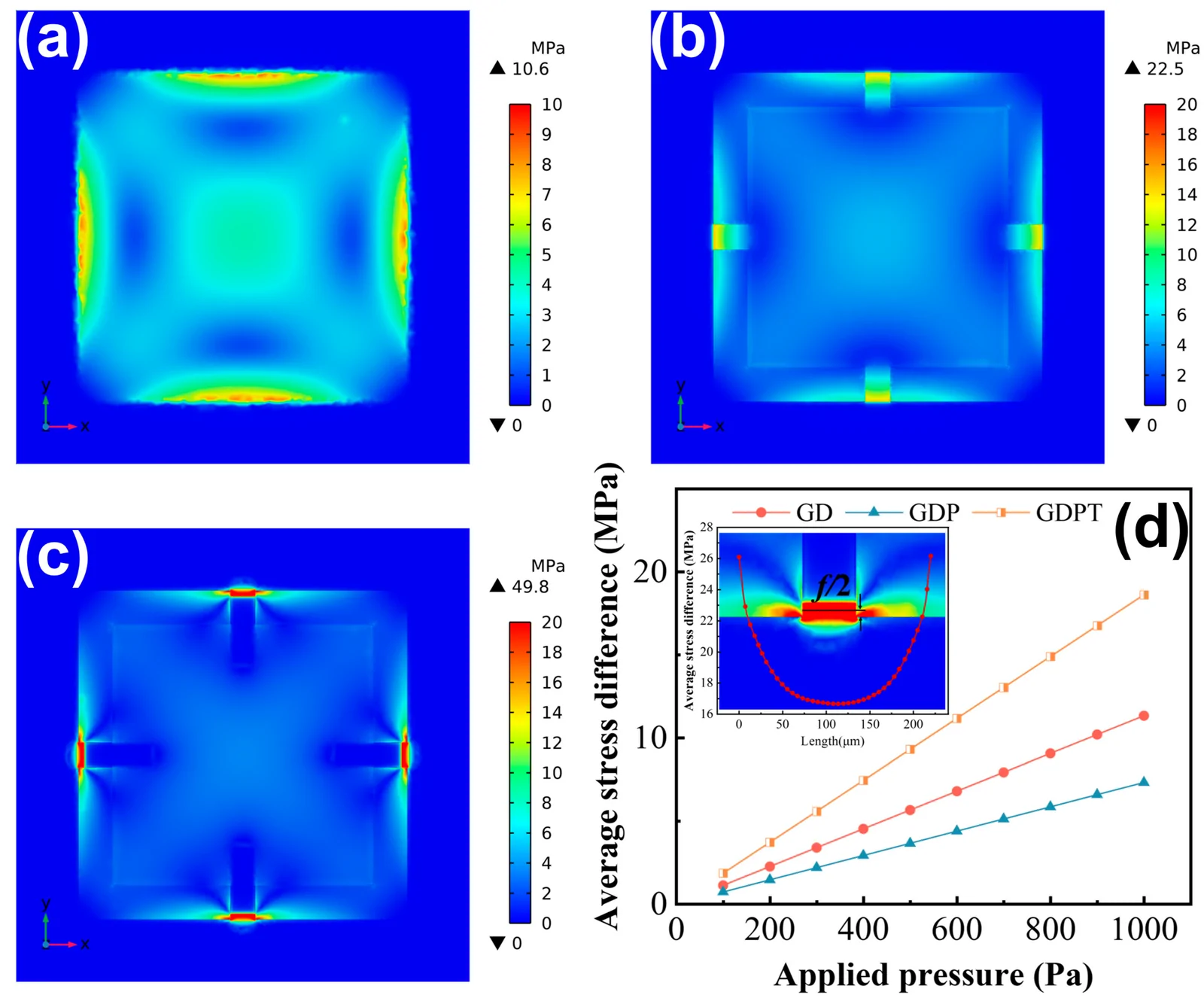
The simulated stress distributions in the (**a**) FD, (**b**) GD, and (**c**) GDPT and (**d**) the average stress difference along the defined path.

To further validate the effectiveness of the new design, FEA simulations are conducted to analyze the sensing behaviors of the FD, GD, and GDPT. The GD is obtained by introducing 300 μm wide, 3 μm deep grooves with 220 μm wide ridges into the 2900 μm × 2900 μm × 15 μm FD. The GDPT further incorporates four back peninsulas with a width of 220 μm and length of 300 μm and corresponding trenches on the frame with a length of 300 μm and the same width as the peninsulas. All diaphragms are modeled as isotropic single-crystal silicon with a density of 2329 kg/m^3^, Young’s modulus of 170 GPa, and Poisson’s ratio of 0.28. Accuracy and throughput are balanced by a custom free tetrahedral mesh, and its density is refined around the geometric singularities and relaxed elsewhere, yielding mesh-independent solutions at a minimal computational cost. In the simulation, the bottom surface of the chip frame is fixed, and the pressures are perpendicularly applied to the front surface of the GDPT. The stress distributions of the three structures are shown in Figure 4. It can be observed that the FD exhibits a large stress distribution region, and the peak stress is concentrated in the central area of the diaphragm edge, without a distinct high-stress region. The GD forms pronounced stress concentration in the ridges, and the peak stress is significantly enhanced compared to the FD. Benefiting from the collaboration of the front grooves and back peninsulas/trenches, the GDPT significantly reduces the dissipation of stress energy and produces significantly higher-stress regions for the piezoresistor. A sampling path is defined to acquire the stress values in the three diaphragms, and the path location is shown in the inset of Figure 4d. The path length is consistent with the width of the GDPT ridge, and the distance from the diaphragm edge is *f*/2. The longitudinal and transverse stresses in the FD, GD, and GDPT are collected along the path, and their average differences are calculated and shown in Figure 4d. It can be seen that the stress under 1 kPa pressure for the FD is 7.32 MPa, and this increases to 11.33 MPa for the GD and reaches 18.61 MPa for the GDPT. The corresponding improvements obtained with the GDPT are 154% and 64% compared with the FD and GD, respectively.

**Figure 5:**
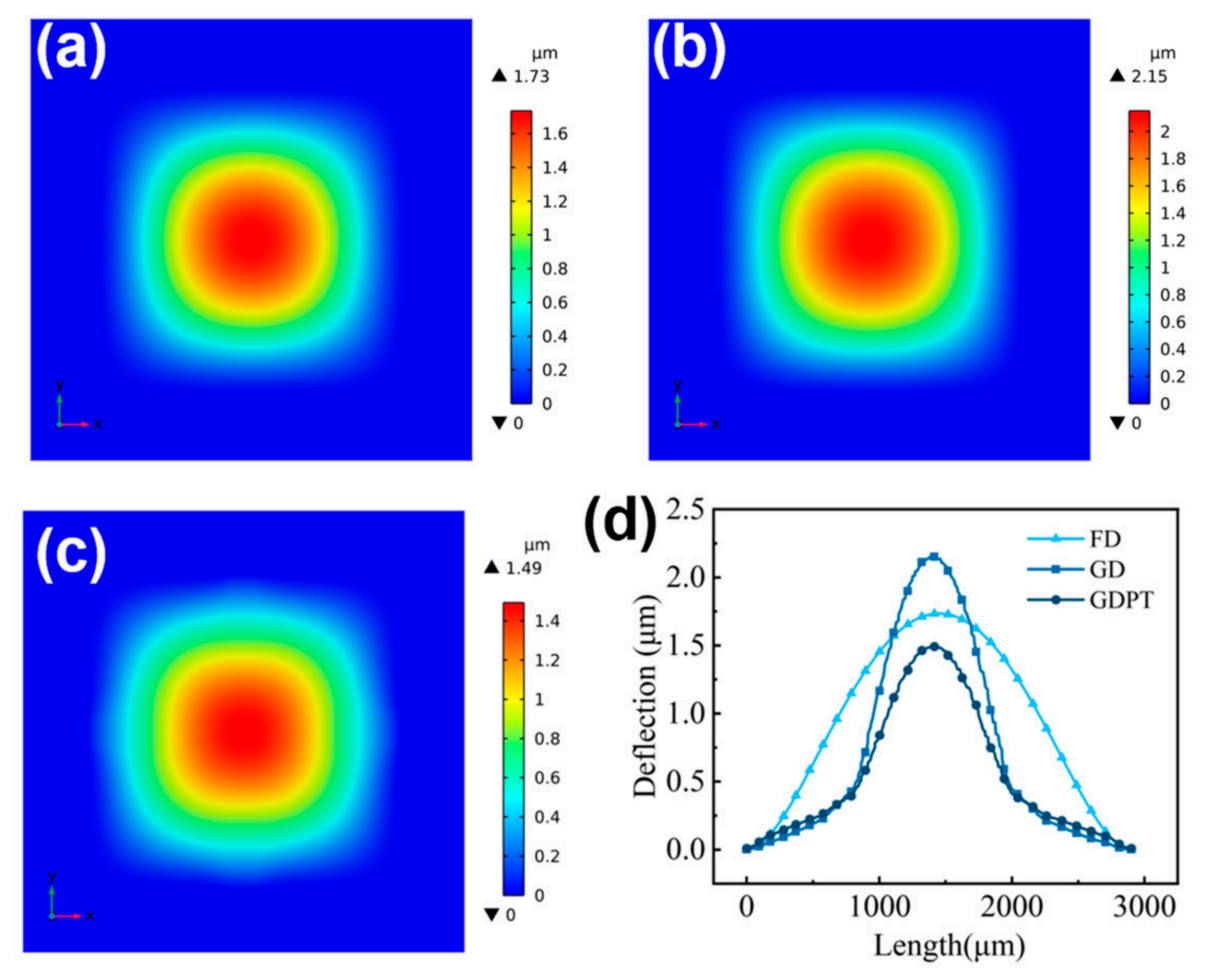
The simulated deflection distributions in the (**a**) FD, (**b**) GD, and (**c**) GDPT and (**d**) the deflections along the diaphragm central line.

The simulated deflections of the three structures are shown in Figure 5. The deformation distribution patterns are essentially identical, but significant differences exist in their maximum amplitudes. The maximum deflection of the FD structure is 1.73 μm, and the value reaches 2.15 μm for the GD due to the edge thinning effect of the added grooves. As for the GDPT, the maximum deflection is only 1.49 μm, representing a decrease of 13.87% and 30.70% compared to the FD and GD structures, respectively.

**Figure 6:**
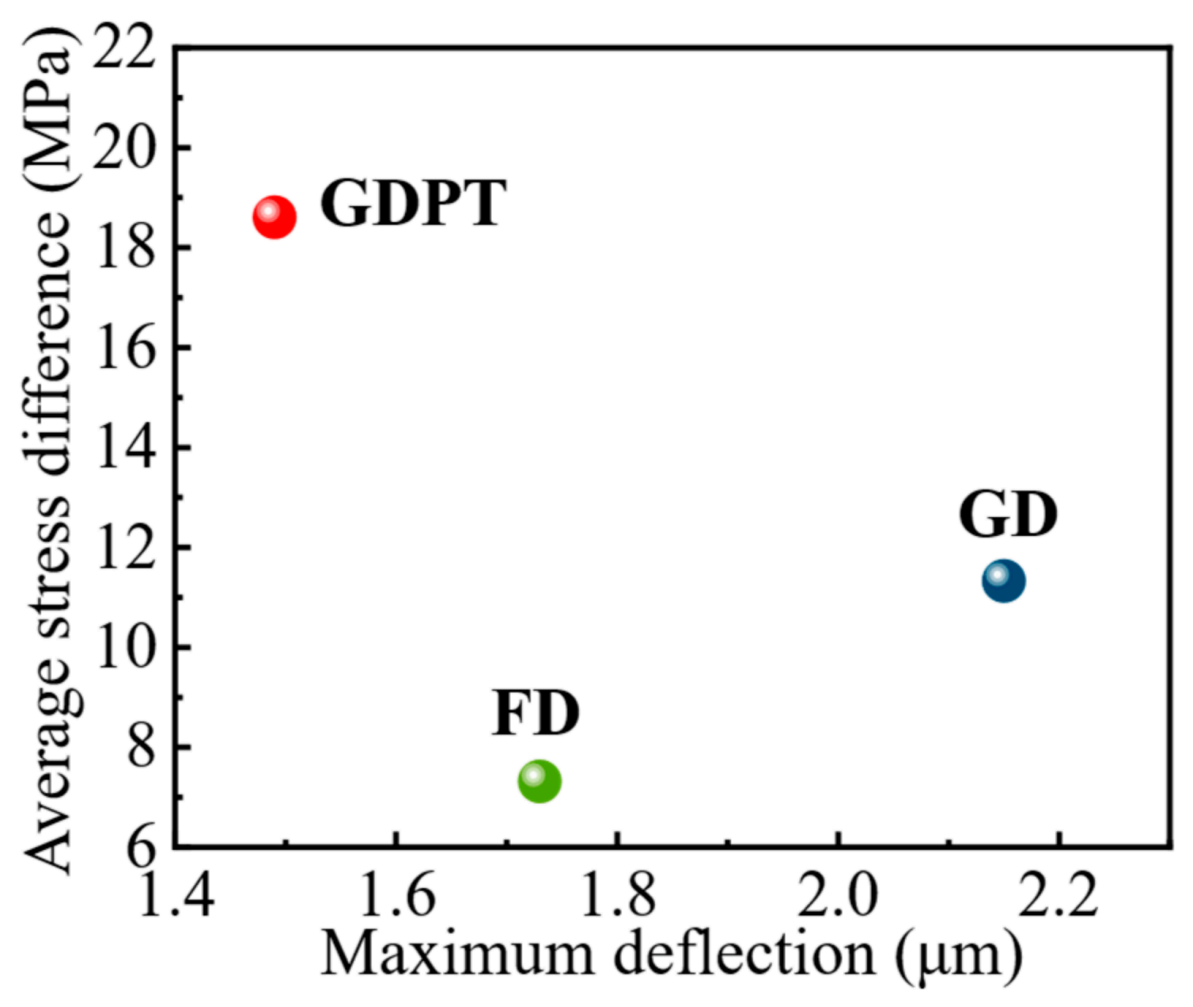
Comparison of the sensing responses of the three diaphragms.

Figure 6 further compares the response behaviors of the three diaphragms. It can be seen that the FD exhibits the lowest sensing stress, while the GD shows improved stress at the cost of the largest deflection. Through its distinctive stress concentration and local stiffness modulation, the GDPT simultaneously achieves the highest stress and the smallest deflection, and it successfully alleviates the interaction between the sensitivity and nonlinearity, demonstrating significant advantages for 1 kPa pressure measurement.

### 2.3. Neural Network-Based Dimension Generation

**Figure 7:**
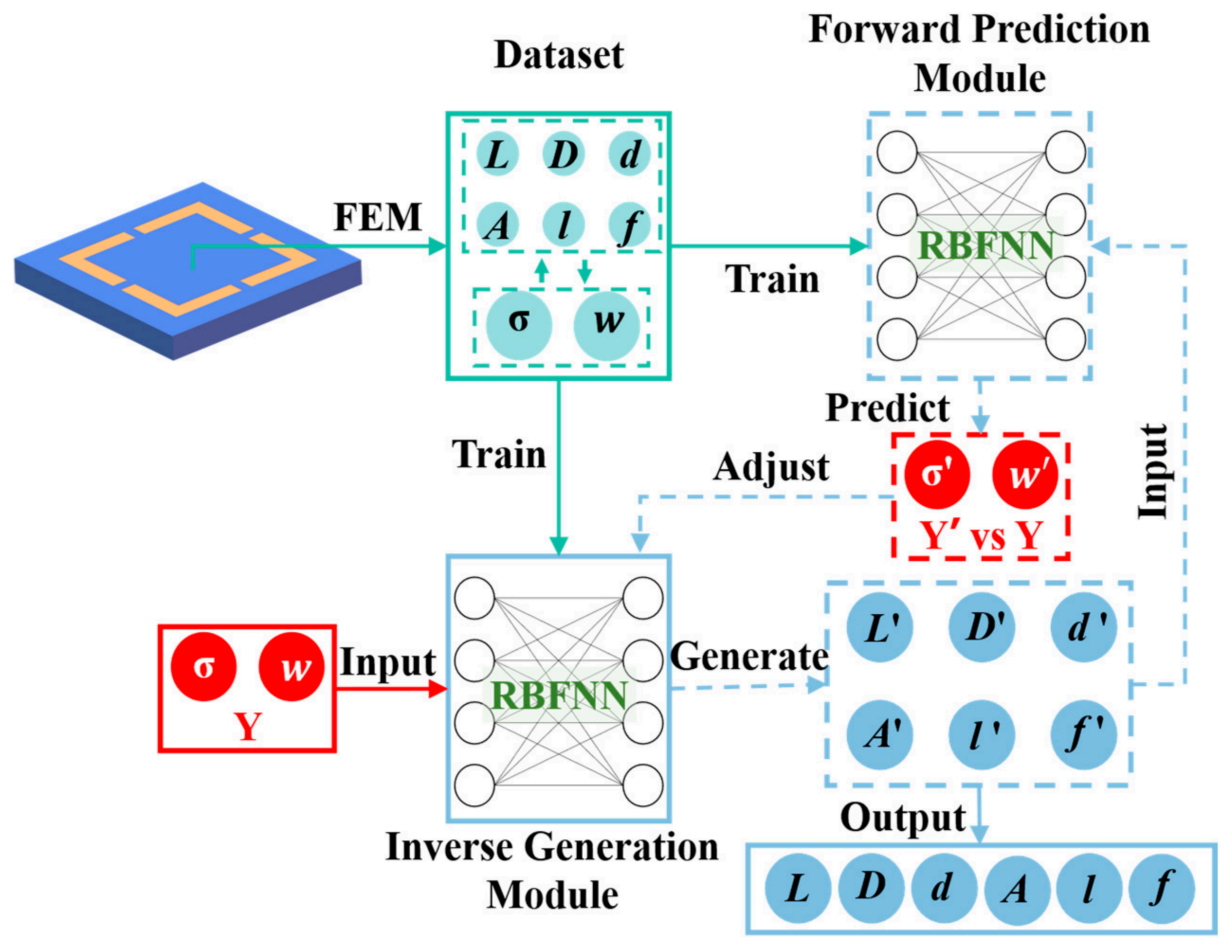
The architecture of the proposed RBFNN-based dimension generator.

The new GDPT has demonstrated promising application potential for 1 kPa pressure sensors. For this type of complex structure, solely using FEM simulation and manual trial-and-error iteration to obtain satisfactory dimensional groups to achieve the expected measurement performance can be a time-consuming process. Additionally, the traditional approaches also suffer from limited generalization capabilities, requiring numerous repeated simulations even for slight adjustments to the performance criteria. This inefficiency impedes the widespread application of the GDPT across diverse measurement scenarios. Considering this issue, we directly utilize the developed RBFNN-based dimension generator to produce practicable dimensions according to preset performance criteria. The main architecture of the generator is shown in Figure 7; it utilizes a forward prediction module (FPM) to map structural parameters (*A*, *D*, *L*, *d*, *l*, and *f* in Figure 1) to sensing responses (average stress difference and maximum deflection) and an inverse generation module (IGM) for sensing-response-to-structural-parameter mapping. The FPM is tandemly connected behind the IGM to prevent data contamination arising from the non-uniqueness problem in response-to-parameter mapping. In generator training, the predetermined targets Y are input to the IGM, and the produced dimensions are passed to the pre-trained FPM to calculate the corresponding response parameters Y′. The deviation between Y and Y′ is utilized to modulate the IGM parameters until convergence is achieved. Both the FPM and IGM are constructed based on the RBFNN, and the PSO algorithm is employed to optimize the initial spread of each module. Detailed information about the RBFNN-based dimension generator can be found in [23].

Taking the full-scale output voltage ≥ 60 mV under 5 V excitation as the sensitivity target for the 1 kPa micropressure sensor, the corresponding average stress difference in the piezoresistive region is calculated to be ≥17.39 MPa according to Equation (2). However, extensive dimension variation is required to provide sufficient datasets for FPM and IGM training, and it is impossible to effectively fix the dimensions of the piezoresistive region. Considering the fabrication process and capacity, the piezoresistors cannot be positioned at the edges of the ridges. Therefore, the stress value from Equation (2) does not align with the requirements of generator training and cannot be directly input to the neural network modules. To address this issue, we employed the confirmable average stress difference along the path defined in Figure 4d to replace the uncertain regional stress as the stress input. To ensure a good degree of matching between the average stress difference along the defined path and the corresponding piezoresistor region, we uniformly sampled 10 different widths for the ridge of the GDPT across the empirically chosen range (150–250 μm) to analyze the ratio of path stress to piezoresistor region stress. The obtained mean ratio (1.441 ± 0.0782) was then used to estimate the input stress for the RBFNN model; thus, the required average stress difference along the path should be ≥25.06 MPa to meet the sensitivity target. Herein, we set this value to 27 MPa, which is an additional design margin introduced to account for the variation in the stress conversion ratios and ensure the reliable achievement of the target. As for the nonlinearity issue, the expected result is compliance with small deflection theory. Considering our existing SOI silicon wafers, their handle layers are all thicker than 10 μm. Correspondingly, the maximum diaphragm deflection is set as 2 μm to effectively constrain the geometric nonlinearity.

The set response parameters (27 MPa and 2 μm) are input into the trained dimension generator, and the produced dimensions are listed in Table 1. Further FEA validation with these dimensions is also conducted to simulate the corresponding responses, and the obtained results are also recorded in Table 1. It can be seen that the newly simulated responses are in excellent agreement with the preset targets at a relative error of 0.85% for the path average stress difference and 0.5% for the maximum deflection. Meanwhile, a statistical analysis based on 140 trials indicated that the entire generation process consumed only 0.67 ms, representing a dramatic efficiency improvement over the conventional FEA trial-and-error methods.

## 3. Fabrication and Characterization

### 3.1. Sensor Fabrication

**Figure 8:**
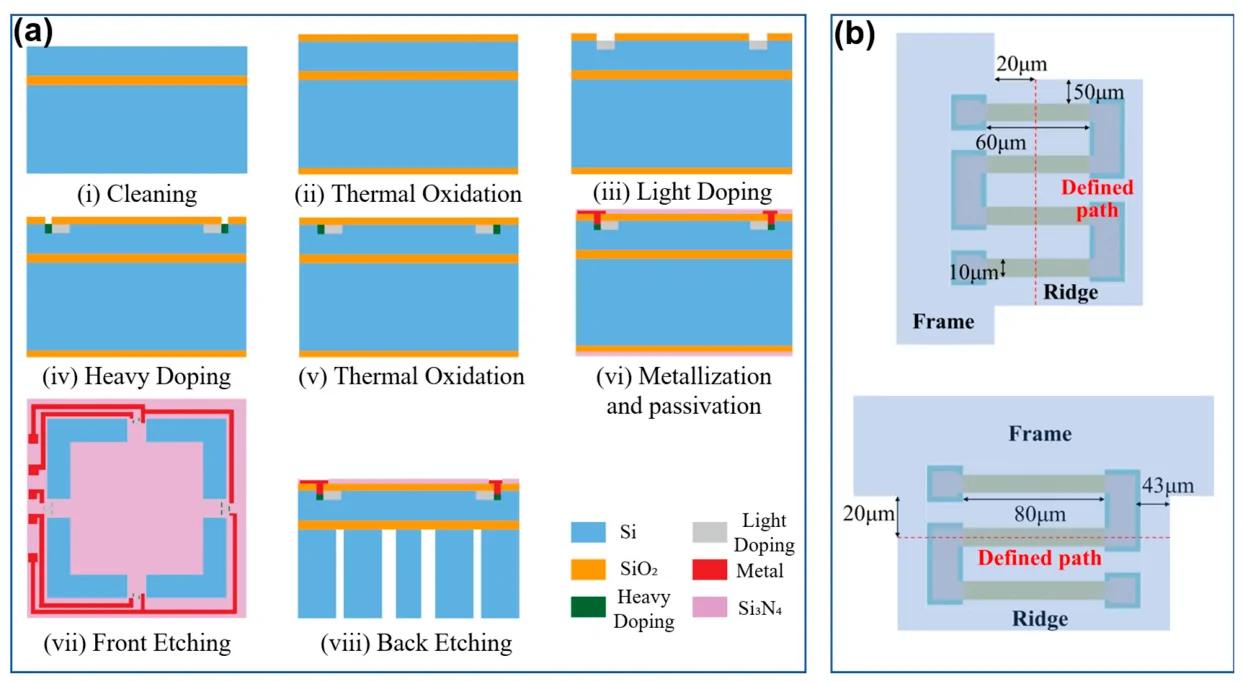
Fabrication of the sensor chip: (**a**) fabrication flow and (**b**) the piezoresistor positions.

According to the dimensions from the generator, a 6-inch n-type (100)-oriented silicon-on-insulator (SOI) wafer with a 15 μm handle layer, 1 μm oxide layer, and 400 μm substrate was selected to fabricate the GDPT sensor chip. Considering the available micro-nanofabrication capabilities, the dimensional parameters derived from the generator were fine-tuned and rounded during the chip fabrication stage (also listed in Table 1), and the rationality was further verified to ensure the achievement of the design targets. This adjustment was performed based on two primary considerations. First, the RBFNN-generated dimensions were incompatible with our microfabrication capabilities due to the decimal values. Second, the manufacturing engineers requested to round all values to the nearest multiple of ten or the nearest hundred. An overview of the fabrication process is shown in Figure 8a. First, photolithography and light boron implantation were employed to pattern and form the piezoresistors on the front side of the wafer, after the SiO_2_ layers were thermally grown on both sides. After the thermal activation process, four piezoresistors were achieved with sheet resistance of about 200 Ω/□. All piezoresistors were symmetrically positioned at the roots of the ridges on the front surface of the chip, with the defined path serving as the other symmetry axis. The detailed layout of the piezoresistors is shown in Figure 8b. Then, heavy doping was conducted on the front side to realize the contact sections and reduce the influence of lateral regions in each piezoresistor. Contact hole etching and sputtering were successively conducted to achieve the required metallization. Four front grooves and a back peninsula–trench structure were, respectively, etched at the front and back sides of the wafer. Finally, a Si_3_N_4_ passivation layer was deposited on the front side of the chip for protection, excluding the bonding pads.

**Figure 9:**
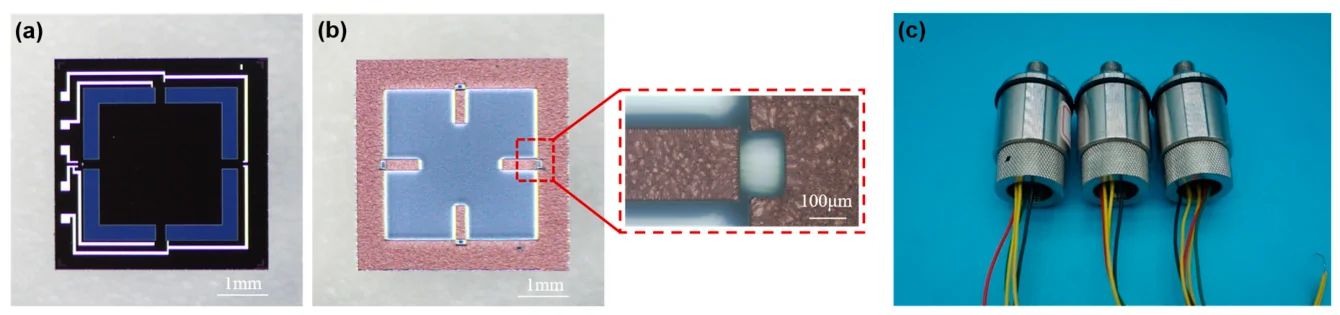
The fabricated sensor chip: (**a**) front and (**b**) back and (**c**) the packaged sensor prototype.

The fabricated sensor chip is shown in Figure 9a,b. Next, a sensor prototype for performance verification was created, and the finished ones are shown in Figure 9c. Herein, the packaging process is briefly presented. The diced chip is assembled onto the sintering base using an adhesive, and the gold wire bonding process is implemented to realize the electrical connection between the pads on the sensor chip and the pins in the sintering base. A via hole is reserved in the packaging structure for loading differential pressure on the backside of the sensor diaphragm. The necessary signal wires, an antileak rubber ring, and a metal shell are subsequently employed to complete the entire packaging process.

### 3.2. Sensor Characterization

**Figure 10:**
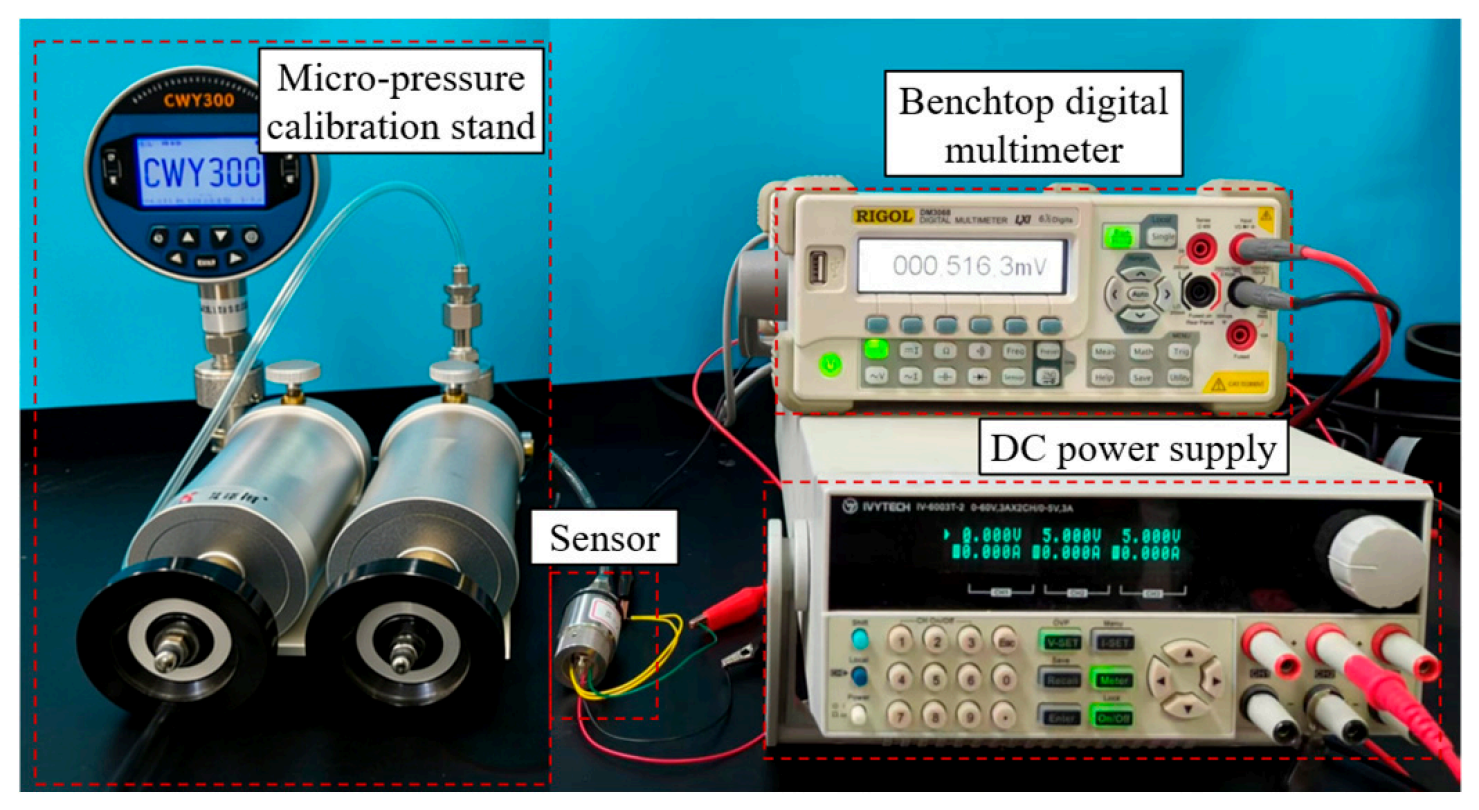
The experimental setup for sensor characterization.

**Figure 11:**
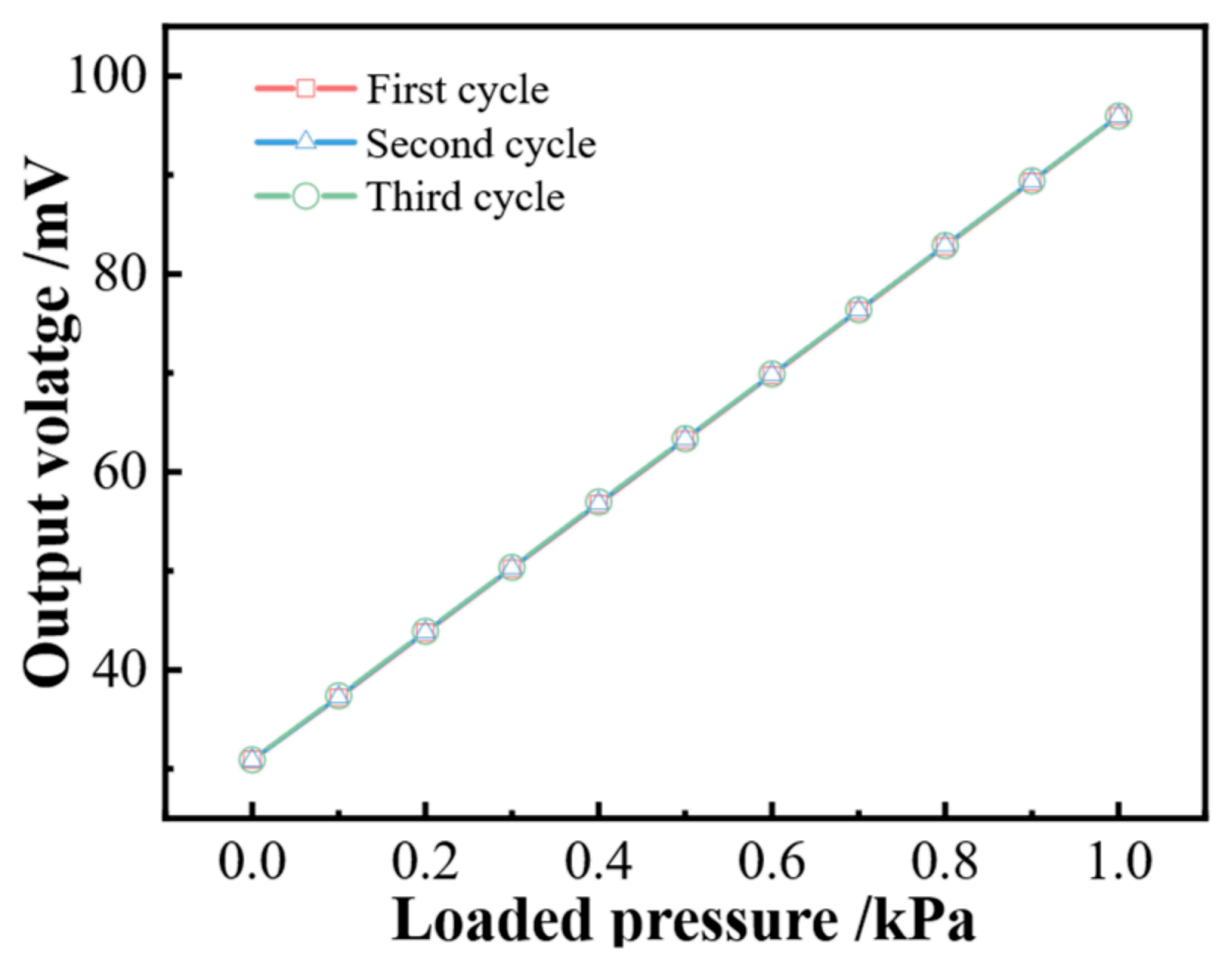
The characterization results for the sensor prototype.

The experimental setup for sensor characterization is shown in Figure 10. The core equipment includes a pressure calibration stand (CWY300, Creat Wit, Xi’an, China) as the standard pressure source, a high-precision DC power supply (IV6003, IVYTECH, Dongguan, China) for 5 V bridge excitation, and a digital multimeter (DM3068, RIGOL, Beijing, China) for sensor output acquisition. Ten calibration points are selected within the 0–1 kPa range, and three loading–offloading cycles are implemented. The obtained input–output results are shown in Table 2 and Figure 11. The test results indicate that the full-scale output of the sensor prototype is 65.05 mV, corresponding to sensitivity of 13.01 mV/(V·kPa). The tested sensitivity satisfies the design target well and reflects that the input stress for the generator is larger than the critical value for the 60 mV case. The measured nonlinearity error is only 0.26% FS. The repeatability and hysteresis errors of the sensor are 0.62% FS and 0.31% FS, respectively. In the characteristic calculation, the least squares method is employed to process the pressures and average outputs in the three loading–offloading cycles. The obtained characteristic line is then used to calculate the sensitivity and the three error parameters. For repeatability, the Bessel formula is employed to calculate the standard deviation for each calibration point. Detailed definitions and calculation methods can be found in the Chinese national standard: Methods for calculating the main static performance specifications of transducers (GB/T 18459—2026) [27].

**Figure 12:**
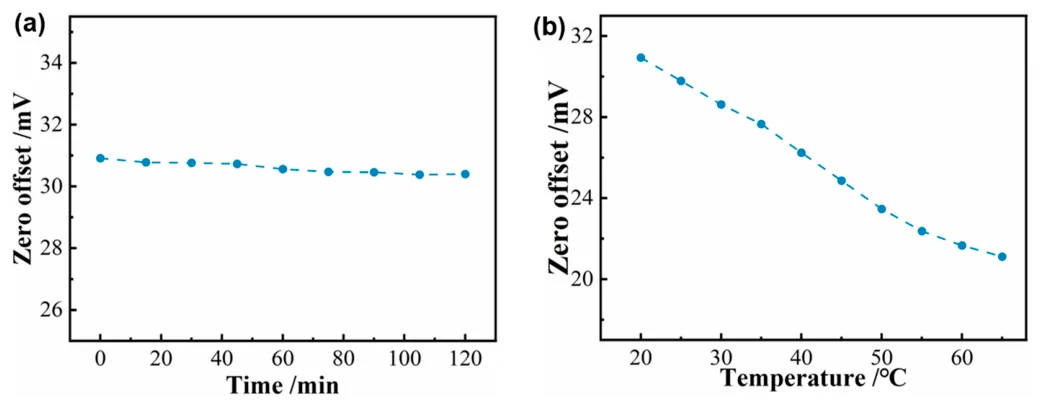
The zero offset drift over (**a**) time and (**b**) temperature.

Due to manufacturing-induced deviation, the four resistors are not ideally matched, and an obvious zero offset is observed in the sensor prototype. Although offset correction has been applied to preclude its influence on sensitivity calculation, additional experiments are still required to evaluate the stability of the zero offset with respect to time and temperature. The obtained results are presented in Figure 12. For the time drift test, the zero offset is recorded at 15 min intervals over a duration of 120 min. The results demonstrate satisfactory stability throughout the test period, with a maximum deviation of 0.38 mV (0.58% FS). For the thermal stability test, measurements are taken over the temperature range of 20–65 °C with 5 °C intervals. At each temperature, the zero offset is recorded after thermal equilibrium is maintained for 30 min. The calculated thermal zero drift coefficient is 0.34% FS/°C, indicating pronounced temperature-induced variation. This behavior is consistent with the general characteristics of piezoresistive sensors and also reveals that the relatively large uncompensated zero offset of the tested prototype may compromise sensor performance in thermally varying environments.

Table 3 summarizes the comparison between our proposed sensor prototype and some other previously published piezoresistive sensors. The sensitivity of the developed sensor clearly surpasses the sensitivities of 4.65–7.23 mV/(V·kPa) observed in recently reported works, and it achieves competitive linearity in the micropressure sensing scenario. The GDPT-based sensor can realize an excellent balance between high sensitivity and low nonlinearity while maintaining a compact diaphragm size, validating its comprehensive performance advantages and application potential. The agreement between the measured and target performance and the demonstrated advantages over existing works validate the superiority of the proposed GDPT scheme in reconciling sensor sensitivity and linearity, as well as confirming the effectiveness of the RBFNN-based dimension generator for rapidly generating feasible dimensional parameters.

### 3.3. Discussion

The primary objective of this work was to apply the GDPT structure and RBFNN-based generator to the development of a 1 kPa micropressure sensor. The favorable characteristics obtained from the experiments validate the advantages of the GDPT and dimensional generator. The objective has been successfully achieved by the obtained sensor prototype in this work. However, compensation for the temperature influence is not covered in this paper. As a conventional challenge faced by piezoresistive sensors, many mature solutions have been proven effective in mitigating temperature-induced effects. Moreover, introducing AI technology into the optimization of the ion implantation dose/energy and sensor calibration can lead to further innovation regarding practicable methods [33,34]. In future demonstration applications, we will also develop intelligent methods to enhance the sensor’s robustness against various environmental interferences. Another concern of this work is that the RBFNN can only generate a single highly optimized parameter set within a continuous-valued range to satisfy the target performance, necessitating additional quantization prior to practical fabrication. This single deterministic solution will restrict the design flexibility to the performance parameters alone, and it fails to adequately accommodate other stages during sensor development. Probabilistic models (e.g., conditional variational autoencoders) can generate a diverse portfolio of candidates under specified constraints and are emerging as more powerful tools for the generative inverse design of micropressure sensors [35].

## 4. Conclusions

In this paper, the effectiveness of the GDPT and RBFNN-based dimension generator in practical sensor development is validated through a 1 kPa micropressure sensor prototype. The GDPT attains excellent reconciliation between sensitivity and nonlinearity, and the generator rapidly determines the key structural dimensions according to preset measurement performance criteria, with the relative deviation not exceeding 0.85%. The sensor prototype is microfabricated, packaged, and characterized for strategy verification. The results demonstrate that the 1 kPa sensor achieves a full-scale output of 65.05 mV under 5 V excitation and a nonlinearity error of 0.26% FS, and it satisfactorily fulfills the design targets. Future work will focus on the strategy’s generalization, design flexibility, and intelligent temperature compensation.

## Figures and Tables

**Table 1:** Obtained dimensions for the 1 kPa pressure sensor.

Parameter	Dimension Name	Symbol	Generated	Fabricated
Dimensional parameter (μm)	Diaphragm length	*L*	3020	3000
	Diaphragm thickness	*D*	14.99	15
	Groove depth	*d*	4.56	5
	Ridge/peninsula width	*A*	190.7	200
	Peninsula length	*l*	624.79	650
	Gap between peninsula and frame	*f*	41	40
Simulated response	Average stress difference (MPa)	-	27.23	27.13
	Maximum deflection (μm)	-	1.99	1.97

**Table 2:** Outputs in three loading–offloading cycles.

Loaded Pressure/kPa	1st Cycle/mV		2nd Cycle/mV		3rd Cycle/mV		Average/mV	RMSD *
	Loading	Offloading	Loading	Offloading	Loading	Offloading		
0	30.99	30.83	30.83	30.89	30.85	30.98	30.90	0.07
0.1	37.24	37.10	37.15	37.36	37.30	37.47	37.27	0.12
0.2	43.67	43.82	43.77	43.88	43.81	43.96	43.82	0.09
0.3	50.2	50.27	50.26	50.38	50.25	50.45	50.30	0.09
0.4	56.69	56.75	56.80	56.88	56.81	57.11	56.84	0.13
0.5	63.23	63.26	63.30	63.39	63.27	63.46	63.32	0.08
0.6	69.73	69.78	69.80	69.88	69.79	70.00	69.83	0.09
0.7	76.27	76.28	76.31	76.42	76.28	76.49	76.34	0.08
0.8	82.79	82.77	82.87	82.92	82.75	83.02	82.85	0.09
0.9	89.27	89.30	89.36	89.47	89.29	89.54	89.37	0.10
1.0	95.83	95.93	95.90	95.90	95.81	96.10	95.95	0.11

* RMSD = root mean square deviation.

**Table 3:** Comparison between our sensor prototype and reported ones.

Work	Range kPa	Sensitivity mV/(V·kPa)	Nonlinearity % FS	Diaphragm Size μm
[26]	6.895	4.48	0.25	4000
[28]	8	5.202 *	0.26 *	3400
[29]	5	6.93 *	0.23 *	2900
[30]	5	5.14	0.28	2900
[31]	10	1.59	0.22	1280
[32]	1	3.401	0.37	1000
This work	1	13.01	0.26	3000

* Value from FEM simulation.

## Data Availability

Data will be made available on request.
